# Whispering-Gallery Mode Resonators for Detecting Cancer

**DOI:** 10.3390/s17092095

**Published:** 2017-09-13

**Authors:** Weeratouch Pongruengkiat, Suejit Pechprasarn

**Affiliations:** Faculty of Biomedical Engineering, Rangsit University, Pathum Thani 12000, Thailand; weeratouch.p59@rsu.ac.th

**Keywords:** optical resonator, whispering-gallery mode, optical waveguide, evanescent wave, label-free, biosensor, cancer, sensor platform, instrumentation

## Abstract

Optical resonators are sensors well known for their high sensitivity and fast response time. These sensors have a wide range of applications, including in the biomedical fields, and cancer detection is one such promising application. Sensor diagnosis currently has many limitations, such as being expensive, highly invasive, and time-consuming. New developments are welcomed to overcome these limitations. Optical resonators have high sensitivity, which enable medical testing to detect disease in the early stage. Herein, we describe the principle of whispering-gallery mode and ring optical resonators. We also add to the knowledge of cancer biomarker diagnosis, where we discuss the application of optical resonators for specific biomarkers. Lastly, we discuss advancements in optical resonators for detecting cancer in terms of their ability to detect small amounts of cancer biomarkers.

## 1. Introduction

**Figure 1:**
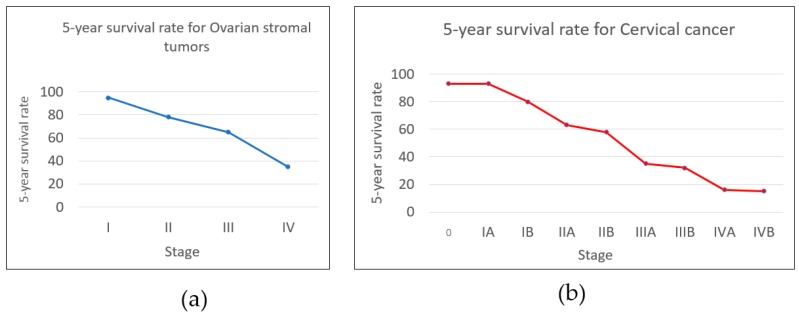
Graphs plotted between 5-year survival rates versus stages of cancers. It is clear that at the initial stage of cancer development, patients have significantly larger chances of being cured. (**a**) Ovarian stromal tumor [5], (**b**) Cervical cancer [6].

Cancer, a hazardous non-communicable disease, is currently the main challenge in healthcare. Cancer Research UK shows that more than 14.1 million people had cancer in 2012 [1]. Despite the growing fatal rate of the disease, cancer develops in stages attributed to different hazard levels; the faster the cancer is detected, the higher the chance it can be cured. Figure 1 shows the survival rates for ovarian stromal cancer and cervical cancer visualized from the 5-year survival rates, which predict the chance of survival for those years [2]. Cancer is usually divided into four stages: Stage I, cancer is small and contained within the organ of origin; Stage II, cancer has grown larger but has not spread to other organs; Stage III, cancer has spread to nearby tissues and can reach the lymph nodes; and Stage IV, metastatic cancer; cancer has spread to other organs in the body. A, B, and C are used to indicate the substage, e.g., lung carcinoid tumor stage IIA [3]. However, the number staging system is an abstraction that describes the disease progression. Healthcare professionals typically describe the disease stage using the tumor-node-metastasis (TNM) system. T evaluates the size of the cancer and its area of spread to nearby tissue on a scale of 1–4; N defines whether the cancer reaches a lymph node on a scale of 0–3; M indicates whether the cancer has spread to another organ, and the value is binary: either 0 or 1 [4]. Table 1 shows the relations between the two systems.

Even now, early diagnoses for cancer are scarce. A qualitative study in 2015 asserted that late diagnosis is the result of difficulty in making appointments, worry regarding doctor availability, and unwillingness to learn of the development of cancer [8]. Apart from the emotional concern of scarring from unfortunate discovery, the findings reflect the difficulty in accessing diagnostic technologies even in developed countries. Currently, cancer detection is still based on highly invasive, time-consuming, and costly processes.

When point-of-care (POC) diagnosis was introduced, the concept of real-time, or at least shorter diagnosis time was heralded as the future of healthcare. The actual definition of POC diagnosis is testing at or near the site of patient care whenever medical care is needed [9]. The first biosensor was a glucose meter that became popular in the late 1980s [10,11]. However, POC diagnosis is not a new concept. At the dawn of civilization, doctors visited patients’ homes and performed diagnosis and treatment without today’s centralized medical centers. The centralized medical complex was introduced in the early 17th century [12], enhancing the mobility of technology and knowledge. Over time, and as the world population increased exponentially, more patients have become dependent on this system. The demographic growth has resulted in an overwhelming demand for healthcare services. The diagnostic and treatment capability are then limited by the capacity of the available technology.

**Figure 2:**
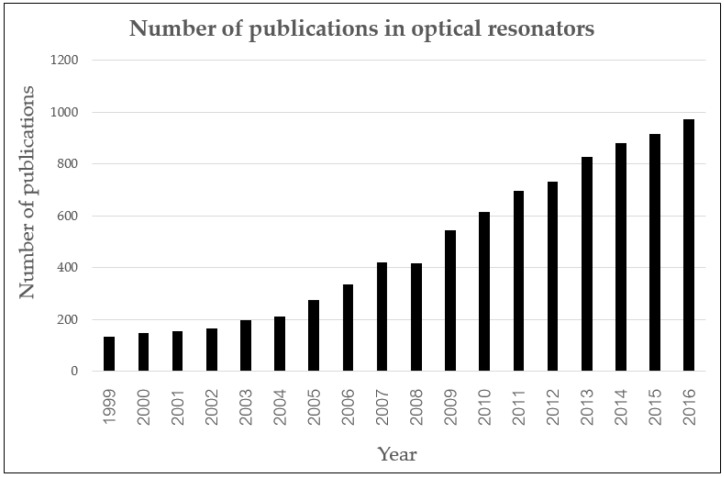
Figure shows how the number of articles on fix in figure label optical resonators evolved between 1999–2016. The graph shows increasing trends in this area of study. Remark: The data were gathered from the Web of Science database (www.web of knowledge.com) with the keyword “Optical Resonator”, and then the category filter “Optics” was applied [19].

Diagnosis requires novel tools and instruments. Based on the concept of reducing diagnostic times and steps, modern biosensors have come to play an important role in fulfilling the ideology of POC. Some cancers now can be detected at an early stage using less invasive and lower-cost procedures [13,14,15] through advancements in sensor technologies such as electrochemical sensor [16], optical sensor, or piezoelectric sensor [14]. This is thanks to the discovery of cancer biomarkers (cancer markers), which have allowed biosensor detection to be more specific [17]. In particular, the optical biosensor has high sensitivity and can perform label-free detection [17,18]. One technique gaining the attention of biosensor researchers is the optical resonator. Figure 2 shows the publications on this type of optical sensor.

Optical resonators enhance light guide properties for detection in the environment. The sensors are based on light confinement in waveguide structures, such as ring resonators, Fabry-Perot resonators, whispering-gallery mode (WGM) resonators (WGR) and high-contrast gratings. Currently, there is high growth in optical resonator research because of their promising properties, such as high sensitivity, low response time, compactness, and immunity to electromagnetic interference [17,18,20,21], which are unlike other waveguide sensors or fiber sensors, in which sensor dimension limits light–matter interaction. Optical resonators determine the interaction length by the quality (Q) factor [22], the dimensionless quantity of temporal confinement of light resonating inside the sensor [23]. WGR and ring resonators are emerging cancer detection technologies known for their easy fabrication and high performance. These aspects allow the sensors to perform early detection when cancer biomarker concentrations are still low. This is the first review of such technologies for cancer detection.

Unlike other sensors, such as electrochemical sensors, which usually require probe labeling or analyte modification [24], optical resonators require no chemical modification of the analyte [21]. Optical resonators can diagnose different cancer biomarkers in a short time [17,25,26,27]. These properties also match the concept of POC diagnosis. Optical resonators have wide-ranging applications. As label-free biosensors, most optical resonators eliminate the pre-detection procedures by removing, or at least shortening, sample preparation time. For label-based sensing, the biological sample must undergo analyte marking, for example, with a fluorescent dye or radio marker. This not only requires extra cost for the labeling material, but also causes undesirable delay between sample collection and analysis. This delay and labeling approach can result in changes in both the physical and chemical properties of the analyte [17,25,28,29,30].

In this review, we introduce the fundamentals of optical resonators and evaluate the various sensors, and describe WGR and ring resonators in more detail. Then, we provide an overview of sensor fabrication and preparation. We briefly introduce cancer biomarkers and discuss their detection. We then review recent works on the application of optical resonators in cancer biomarker detection focused on early stage detection. We also discuss how the high sensitivity of the optical resonators plays an important role in the early stage cancer diagnosis and how these optical technologies can potentially save lots of lives.

## 2. Fundamentals of Optical Resonators

### 2.1. Performance Parameters of Optical Resonators

Similar to other biomedical sensors, optical resonators are defined by sensitivity, limit of detection (LOD), resolution, dynamic range, and selectivity. Sensitivity describes the change in output upon a change in the physical properties of the sensor. For optical resonators, sensitivity is the ability to transduce the change (binding) on the resonator surface into an output, i.e., spectral shift. This can be described as the shift of resonant wavelength (δλ) or the difference of light intensity (δI) at a particular wavelength when the analyte is bound to the surface. The units are usually given as nm/RIU (wavelength shift over refractive index unit) and W/(m^2∙^RIU) [31].

LOD is the minimum quantity of analyte to be detected by the sensor in the actual detection environment. The LOD is limited by noise source, efficiency of the optical detector in the optical system, and the amount of light that can be detected [32]. Optical system noise can come from: (1) thermal noise, (2) microphone vibrations (3) dark current of the detector and (4) shot noise of the optical detector and loss due to material defects. These amounts of noise will introduce losses in the optical resonator [33,34,35]. The LOD for optical resonators is normally defined by two parameters: First, the RIU and surface coverage, given as pg/mm^2^, or it can be described as sample concentration in the molar unit. Label-free sensors are normally described by RIU, while surface coverage/sample concentration are used in both labeled and label-free sensors [36]. RIU can be converted to other physical parameter of analyte by using response unit. In optical sensors, it has been well established and validated over a wide range of different proteins that one response unit of 10^−6^ RIU is equivalent to 1 pg/mm^2^. By knowing the sensing area size, the mass on the sensor can be determined. Similarly, with a known molecular weight, one can determine the number of molecules on the sensor [32].

Selectivity describes the ability to distinguish between the desired analyte and others in the environment. Whenever the analyte does not specifically bind to the sensor, it results in errors to the output signal. Biosensors are now equipped with biologically selective species such as antibody-antigen [37] or enzyme-substrate [38]. However, synthetic substances have also been developed; aptamer is one such example [39]. The process of introducing the binding material to the resonator surface to optimize selectivity is termed surface functionalization.

Unlike other sensors, biomedical sensor development aims to reduce the sample volume, especially when the analyte requires invasive extraction procedures from a patient, such as a blood sample. The other parameter is the Q-factor, introduced earlier as the basic parameter for determination of the lifetime of light resonating in the waveguided resonator. The Q-factor can be defined as:

(1) $$Q=\omega_{0}\tau =2\pi v_{0}\tau =\frac{\omega_{0}}{\Delta \omega_{FWHM}}$$

where *ω*_0_ is the angular frequency. The linear frequency of the mode is described by *v*_0_. *τ* is the time for the field intensity to decay by the factor of *e*, the so**-**called cavity ring down lifetime. According to the equation, **∆***w_FWHM_* is inversely proportional to τ and determines the line width: the uncertainty of the frequency of resonance in angular frequency; FWHM stands for full width at half-maximum. The highest Q-value reported so far is 2 × 10^10^ on the spheroidal crystalline WGMR with a resonant wavelength of 1300 nm [40]. For amorphous material, it is 8 × 10^9^ with a 633 nm resonant wavelength [41].

### 2.2. Light Coupling

Light coupling is commonly referred to as evanescent wave coupling in optical resonator research. It can be described as inducing light on another medium without contact. As two optical guide components are placed within evanescent zone distance, light is coupled into the resonant structure aided by a phase-matched evanescent field. A tunable laser source is commonly used for input; the wavelength of the source can be adjusted. At the matching wavelength, the intensity dip can be observed using a photodetector. As mentioned earlier, the optical resonator interaction length is the effect of a Q-factor. The length is described by:

(2) $$L_{eff}=\frac{Q\lambda }{2\pi n}$$

From Equation (1), *L_eff_* is the effective interaction length and *n* is the refractive index of the resonator. Q-factors of typical ring resonators range from 10^4^ to 10^9^. *λ* is resonant wavelength; the matching wavelength is determined by the resonant condition:

(3) $$\lambda_{m}=\frac{2\pi rn_{eff}}{m}\;\mathrm{for}\;m=1,\;2,\;3,\;\ldots$$

where *r* describes the outer radius of the ring resonator; *n_eff_* refers to the effective refractive index, which is sensitive to the binding event on the surface; m is the mode number; *λ_m_* is the resonant free-space wavelength of the tunable laser.

**Figure 3:**
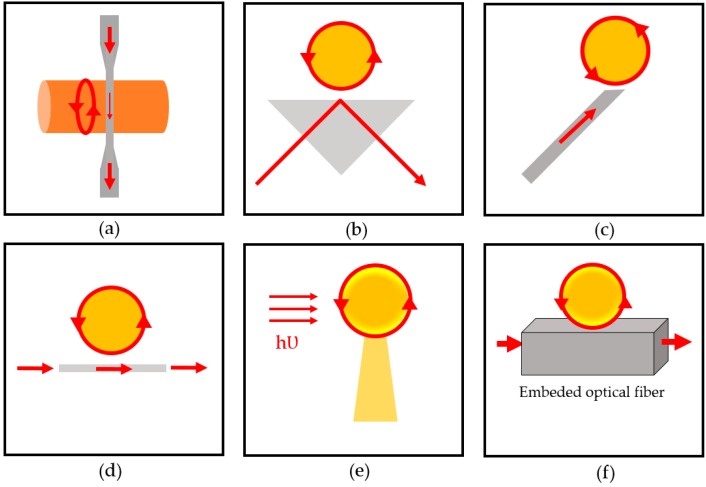
Various types of optical coupling. (**a**) Tapered; (**b**) prism; (**c**) angled fiber; (**d**) planar waveguide side; (**e**) free-space; (**f**) polished half-block coupler.

Optical resonators mostly consist of two optical waveguide structures: the first serves the system as an input and output waveguide, where light enters and the signal is detected at the other end. The other structure is the resonator structure, which confines the light propagated from the first structure. There are various techniques for coupling the light, the most common being tapered coupling. Examples of methods for illuminating resonator structures (Figure 3) include tapered coupling [42], prism coupling [43], angled fiber coupling [44], planar waveguide side coupling [39,45,46], free-space coupling or direct illumination [47,48], and polished half-block coupler [49].

Tapered coupling has 99.8% coupling efficiency [50]; the losses are the result of material absorption, scattering, and bending losses from fiber stress. Comparable efficiency has been reported for the half-block coupler [49]. Experimental studies have shown that prism coupling has approximately 80% efficiency [51,52]. Angle-polished fiber coupling, also known as “pigtailing,” has 60% efficiency [44]. Tapered coupling has the advantages of not only higher coupling efficiency but also ease of fabrication and preparation. Despite their lower coupling efficiency, the other coupling methods are of research interest, as they can provide more robustness [18].

### 2.3. Whispering-Gallery Mode

Lord Rayleigh discovered WGM in 1878, as he whispered on one side of the curved wall inside St**.** Paul’s Cathedral (London, UK). His voice could be heard 40 m away [53,54]. This demonstrated the phenomena of the acoustic wave (sound), which could travel along the edge of the gallery hall with negligible loss. As he observed this phenomenon, he also proposed that electromagnetic waves could travel with this mode. In 1961, the WGM of optical light was first reported in a spherical microstructure [55]. Instead of the curved wall-guided whispered voice, the light was entirely internally reflected in a confined cavity. Later on, WGMs in liquid were also studied. Following Lord Rayleigh’s discovery, Debye and Mie published two important theoretical works. Debye determined the resonant eigenfrequencies for dielectric and metallic spheres in 1909 [56]. Mie studied electromagnetic wave scattering in microspheres [57]. These later became widely discussed in both theoretical and experimental works [53].

**Figure 4:**
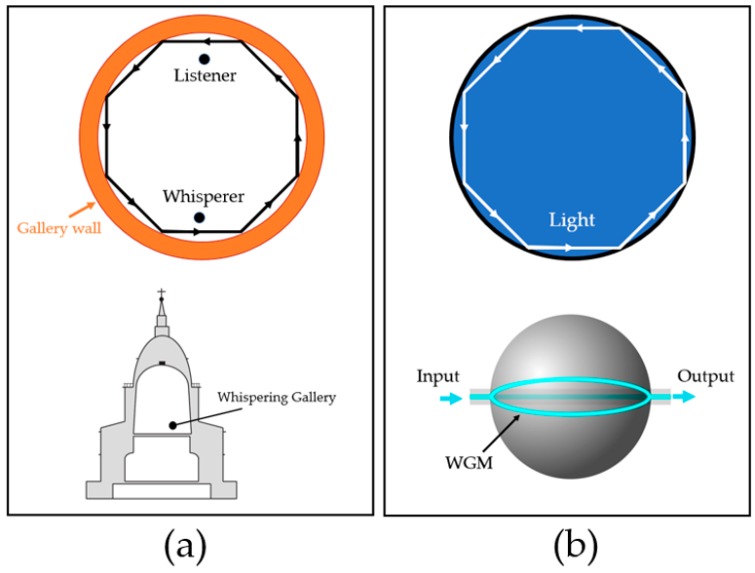
Comparison of mechanical wave and electromagnetic wave WGM. (**a**) Diagram shows how WGM occurs: sound travels from one side of the gallery to another in the structure of St. Paul’s Cathedral where WGM was first observed; (**b**) WGM of light: light is totally internally reflected within the small curved cavity [54,75]. The phenomenon can be observed as light trapped inside the spherical structure [76].

Optical WGM, as mentioned earlier, was discovered in a microsphere resonator. Crystalline calcium fluoride (CaF_2_) was fabricated as the resonator. A pulsed laser was illuminated in a tangential direction to the sphere. The detected output laser showed transient oscillation instead of spikes from the input, confirming the presence of WGM [55]. In 1981, WGM in liquid resonators was observed for the first time. In the experiment, a liquid droplet was optically levitated by a laser beam, and the scattered light was detected [58]. WGR have many optics and optoelectronics applications and are studied in both passive and active mode [59]. The previous applications for passive mode include optical and photonic single resonator filters [60,61], high-order filter or cascade resonators [62,63], tunable filters [64], WGM filters in optoelectronic oscillator (OEO) [65,66], and sensors, which can be biological, chemical, or mechanical [67,68,69,70]. The active-mode WGM is commonly utilized as a laser source, and involves wave mixing devices such as continuous-wave (CW)-WGM laser, i.e., the miniature laser [55,71], resonator-modified scattering [72], switches and modulators [73], OEO [74], pulse propagation and generation, and wave-mixing oscillator [59]. The comparison of WGM of sound and optical light is shown in Figure 4.

### 2.4. Detection Mechanism

Total internal reflection generates an evanescent wave on the resonator surface. This allows the resonator to detect any analyte in the environment bound with the resonator. Fundamentally, optical resonators are sensitive to the refractive index. The analyte molecules binding to the resonator surface cause a shift in the effective refractive index [33,77]. The measurement is processed by plotting the graph between light intensity versus wavelength, i.e., the so-called spectral shift. The surface molecule density is related to the spectral shift and can be described by the first-order perturbation theory [68,78,79].

(4) $$\frac{\delta \lambda }{\lambda }=\frac{\alpha_{ex}\sigma }{\varepsilon_{0}(n_{ring}^{2}-n_{buffer}^{2})r}$$

**Figure 5:**
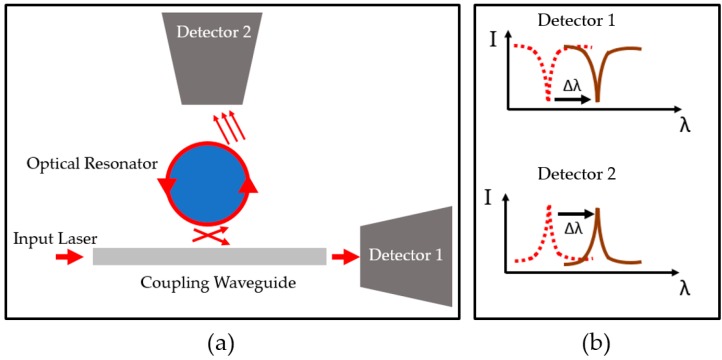
Diagram shows the optical resonator system and analysis. (**a**) Optical resonator system: the laser guided by a coupling waveguide excites the resonator. The detector is equipped to measure the intensity dip of the resonant wavelength at the end of the coupling waveguide (Detector 1) or scattered light (Detector 2). (**b**) Graph shows the absence of light at resonant wavelength. When the analyte binds on the surface, it causes a wavelength shift that can be observed by both modes.

Equation (3) reveals the relationship between molecule surface density (σ) and spectral of microring resonator, where *λ* is resonant wavelength and *δλ* is the shift of resonant wavelength; *ε_0_* is constant vacuum permittivity, α_ex_ is excess polarizability for molecules; *n_ring_* and *n_buffer_* are the refractive index of the microsphere resonator and buffer solution, respectively, while *r* is the ring radius. Figure 5 depicts the optical resonator system.

## 3. Optical Resonator Types

Optical resonator types are defined by their geometries and materials. The examples of geometries range from the heavily used mirroring [37,67,80,81,82] to microspheres [27,83,84,85], microgoblets [86], disks [21,80,87], microbubbles [88,89], microtoroids [90], and bottles [20]. Resonators generally utilize dielectric material. However, in recent research, there is increasing interest in polymer-based structures. Polymers are used to expand the materials options that might be compatible with other systems such as electronics, and polymers can be manufactured more easily than typical silicon-based structures [80,82,91]. Optical resonators can also integrate with other biosensing systems such as microfluidics [81] or lab-on-a-chip (LOC) [92]. The main types studied in biomedical applications, especially for cancer detection, are configurations based on microring [17,92,93,94,95] and spherical optical resonators [27].

Optical resonators or optical resonant sensors are evanescent wave-based sensors [36], which can be fabricated as a microstructure with different geometries. The sensors trap light inside their microcavities, allowing the optical rays to resonate in the confined space aided by light coupling via optical waveguides. Optical resonators are used not only for biomedical detection [21,96,97] but also for gas detection [98], environmental control [87], toxin detection [99], temperature detection [70,100], microforce detection [67], single protein detection [90] and even nanoparticle detection [101,102].

### 3.1. WGM Microsphere Resonators

**Figure 6:**
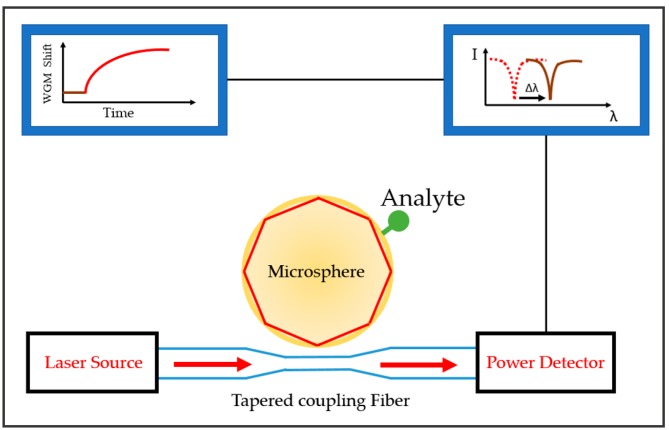
Example of microsphere configuration. Shown is an example of a microsphere with tapered coupling. The microsphere utilizes the WGM principle to resonate light inside its cavity. The analyte binding on the microsphere surface causes the change in the refractive index. The resonant wavelength is also shifted as a result.

These three-dimensional (3D) resonators are typically fabricated by introducing heat to one end of the optical fiber, which will melt the fiber. The molten material soon forms a spherical shape with the aid of surface tension. The sphere has low surface roughness, helping the sensor achieve dramatically high Q-factors in the range of 10^6^ to 10^9^. The sensor has a very low LOD: 10^−8^ to 10^−9^ RIU [68,78]. The configuration is utilized in miniature scale detection, down to the single molecule. Figure 6 depicts the setup.

Unlike ring resonators, the 3D geometry of a microsphere resonator means it has various methods for coupling, i.e., tapered coupling, prism coupling, angled fiber coupling or even direct illumination. Prism coupling and half-block coupling have the advantage of illuminating multiple microsphere resonators simultaneously [27].

Microsphere resonators are also considered easy to fabricate. As mentioned above, the principle is based on melting a waveguide fiber and allowing the surface tension to reform the material into a sphere. The heat applied to the tip of fiber can be flame or an electric arc. The melted fiber, e.g., silica (SiO_2_), will need to retain a minimum value of surface energy, forming a sphere. Then, the material solidifies after the heat source is removed. Despite the simplicity of the fabrication, microsphere formation has low reproducibility. The fused fiber is very sensitive to the environment and contamination. Although the sphere size can be adjusted by selecting the desired size of the preheated fiber, some errors still occur during experiments [103].

### 3.2. Ring Resonators

#### 3.2.1. Microring and Microtoroid Optical Resonators

**Figure 7:**
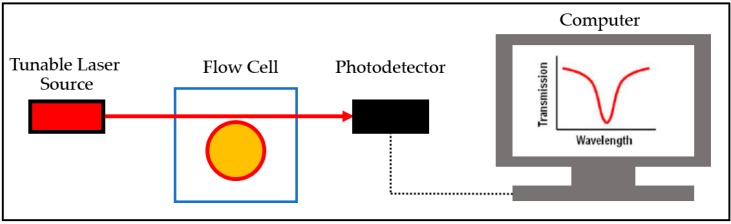
Diagram shows an example common microring optical resonator setting. A tunable laser source provides the optical input through the coupling waveguide. The coupling illuminates the resonator structure. The coupling wavelength can be observed using a photodetector and an analytic instrument such as a computer. The wavelength shifts when the resonator is bound with the analyte, altering the refractive index.

The term “microring resonator” often refers to a planar ring resonator, the configuration being a microscale waveguide in a circular geometry (Figure 7). This resonator has the advantage of ease of fabrication [82]. Silicon or silicon nitride is commonly utilized as the resonating structure. The resonator and coupling waveguide can also be fabricated on the same substrate, meaning all structures are on the same chip. The ring diameters range 10 μm to 10 mm [78,87,92,99,104]. Microring resonator arrays can also be fabricated and have been commercialized. Genalyte (San Diego, CA, USA) manufactures microring resonators that can perform 128 tests within 15 min [105]. There are three main approaches to the fabrication process. The first is deep ultraviolet (DUV) lithography, the main fabrication technique for complementary metal-oxide semiconductor (CMOS). The resolution is comparably rough to other techniques (100 nm). The UV wavelength is 248 nm or 193 nm. The second method, electron beam lithography (EBL), has higher resolution in the range of 10 nm. This method creates fewer flaws than DUV, the trade-off being the longer fabrication time. Lastly, nanoimprinting lithography (NIL) requires pre-processing from the earlier two techniques. The polymer is applied to a mold, and then cured to solidify. The mold is later utilized to create the replica of the structure with waveguide materials. The polymer mold itself can also be the resonator [106].

The resulting Q-factor of ring resonators use to be comparably low (10^4^) [62,107] against microsphere and microtoroid configurations. This is the result of residual flaws during the microfabrication. The other reason is because optical wave leakage occurs as the resonator is connected to the substrate [58]. The surface roughness of the device is often generated during the fabrication process. This considered as defect which lead generate noise, resulting in lowering the quantity of Q-factor as discuss in previous session. However, ultra-high Q ring resonator was discovered later. The modification of the direction coupling waveguide to the resonator instead of a traditional single straight bus waveguide [108,109].

**Figure 8:**
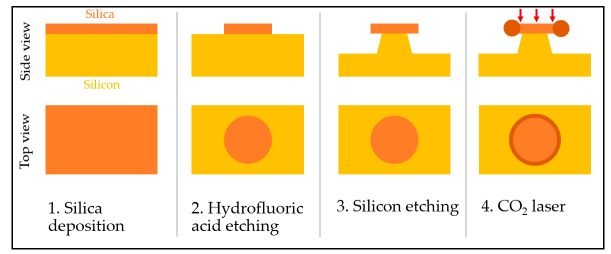
Microtoroid resonator fabrication process. (**1**) SiO_2_ is deposited on a silicon wafer; (**2**) Hydrofluoric acid etching is applied to create the disk structure on top of the wafer; (**3**) XeF_2_ etching is used to create a post structure; (**4**) CO_2_ laser illuminates the structure to smoothen the toroid structure.

Microtoroids, on the other hand, were invented to address the signal lost problems of ring resonator. Even though, microtoroids are WGM-based optical resonator, the device is fabricated on a chip like in microring resonator fabrication. The microtoroid structure is raised above the substrate by the post, preventing any leak from evanescent scattering to the substrate [110]. As a result, a high Q-factor can be observed, with values up to 10^8^ [23]. Microtoroids can be fabricated using lithography, reactive ion etching, and xenon difluoride (XeF_2_) etching. Hence, an array of microtoroids can be fabricated. The fabrication process is as follows: The resonator substrate, SiO_2_, is deposited on a silicon substrate. Then, etching is used to create the SiO_2_ disk. The post is created by removing the substrate below the disk via XeF_2_ isotropic etching. Finally, the CO_2_ laser illuminates the structure, and the edge of the disk melts and forms a smooth toroid aided by surface tension [29,111,112] (Figure 8).

The smoother surface generates less noise due to the surface roughness. However, microtoroid has a difficulty of coupling since the resonators are perched atop of silicon pillar, resulting in difficulty of coupling alignment [113,114]. In addition, during the process of CO_2_ laser illumination melt the resonators, the diameter of microtoroid is shrink. This lead to difficulty of monolithically integration for a micortoroid resonant cavity with an on-chip waveguide [113,115].

#### 3.2.2. Optofluidic Ring Resonator (OFRR)

OFRR is also a ring resonator configuration. The resonator is used to overcome the disadvantage of low Q-factor in microring resonators and the low reproducibility of microsphere resonators [116]. The OFRR uses a microscale SiO_2_ capillary with a diameter in the range of hundreds of micrometers. However, the wall thickness can be thin, i.e., <4 μm. This can be considered a parallel microring resonator combined with the fluidic channel. Fiber manufacturing methods are equipped to fabricate such resonators, and reproducibility is enhanced due to the qualified manufacturing process [117], such as capillary pulling or fiber pulling tower. The resonator structure is then conjugated with the coupling device. The Q-factor of such a device is in the 10^6^ range, with a detection limit of 10^−7^ RIU.

## 4. Pre-Processing

### 4.1. Sensor Surface Functionalization

To prepare sensors for detecting the analyte, the resonator surface must be positioned on the specific recognition area to achieve high selectivity [18]. Receptors for a specific analyte are introduced to the system to convert only specific recognition events into the signal. For biomedical application, biological or chemical receptors are immobilized on the sensor surface. Receptor immobilization is a critical step in fabrication for achieving high-performance detection. The crucial characteristics for immobilizing biomolecules are high selectivity, long-term stability, and efficient functionality.

There are various, well-defined methods of surface functionalization, such as physical adsorption [118], covalent binding [119], non-covalent binding [38], or His tagging [120,121]. Physical adsorption means the interaction is based on hydrophobic and electrostatic properties. Although this is the easiest process, its disadvantages are some desorption of the receptor under specific conditions, and low reproducibility.

Covalent binding introduces molecular chemical groups to the resonator surface. Herein, linkers are used to immobilize the receptor. The process is more reproducible; for example, the binding of proteins can utilize thiol, amino, and carboxylic groups. Non-covalent binding requires an active layer on the surface, such as biological affinity binding. Such surfaces are equipped with biological/chemical-specific affinity pairs [21], for example, antigen and antibody [27].

Surface functionalization begins with surface activation. Silanization, involving several silanes (methoxy- and ethoxysilanes) with different functional groups, is the common method of chemically activating silicon, SiO_2_, or silicon nitride. Silanes assist the process by forming strong bonds between organic and inorganic molecules. A coupling agent stabilizes the hydroxy group on the resonator surface by turning them into oxane bonds.

### 4.2. Sample Preparation

For biomedical detection, sample collection, preparation, and preservation are crucial steps prior to detection. Delay can cause changes in physical and chemical characteristics. Beginning with extraction, the desired analyte is isolated from the buffer solution. Isolation efficiency is mainly dependent on the solubility of the analyte and the matrix effect. Examples of such processes are Soxhlet extraction [122], ultrasonic extraction [123], supercritical fluid [124,125], accelerated solvent [126], and microwave-assisted methods [127]. Sample preparation is considered the major bottleneck of the analysis. Most extraction methods require long operation times. Moreover, there is also the high risk of contamination, resulting in errors in analysis. However, in microscale, fluids always behave as laminar flow, which cannot occur on the macroscopic scale. Pressure-driven flow, capillary-driven flow, osmotic flow, and Marangoni flow enhance the transport phenomena. In the microchannels, different fluids flow separately in a more orderly manner, resulting in the fluids being more difficult to fuse to one another.

Microfluidic systems have been introduced and combined with optical resonator systems. Microfluidics are mostly chip-based technology for manipulating and controlling small volumes of fluids, which enables the use of small amounts of patient sample [103,128]. Given the high Q-factor of the resonator, the sensors can perform well with low volumes of analyte. The sensor can be placed in the microfluidic channel designed specifically for the analyte [129]. Thus, sample preparation and analysis are integrated, enhancing significantly higher throughput than a process requiring separate sample preparation, e.g., micro total analysis system (μTAS) and LOC.

Gene-related detection often uses polymerase chain reaction (PCR) to replicate analytes to detectable levels, as most of the detection in this application is known for extremely low target concentrations compared to background molecules. The challenge of optical resonator detection also focuses on how the process can be speeded-up by reducing the gene amplification time by eliminating PCR from the system or using other on-chip methods [38,130,131].

## 5. Application of Optical Resonators for Detecting Cancer

### 5.1. Cancer Biomarkers

According to the Food and Drug Administration (FDA), a biomarker is a characteristic that is objectively measured and evaluated as an indicator of normal biological processes, pathogenic processes, or biological responses to a therapeutic intervention [132]. Different biomarkers can identify various cancers [15] (Table 2). Cancer is a complex disease; its biomarkers may be multiple parameters. Biomarkers enable doctors to define clinical problems at the early stage with precise prognosis and are less invasive for the patient [133]. Body fluids are more promising as biomarkers; however, they always contain other background molecules, or even cells.

Cancer biomarkers are usually obtained from the primary tumor or body fluids. Extracting a marker from the primary tumor might involve a complex and invasive process, particularly when the cancer has metastasized. Other than optical resonators, cancer biomarkers can also be detected through various other processes. The most common tests are enzyme-linked immunosorbent assay (ELISA), multiplex ELISA, and multiplex arrays. ELISA is the most stable technology at present, and was first developed in the 1970’s [134] as a radioimmunoassay (RIA). The main disadvantage of ELISA is that performance depends heavily on antibody quality, the manufacturer, and requires a skillful operator.

In Table 2, cancer biomarkers can be classified into various categories. They can be a gene, antigen, enzyme, or even a cell physical dysfunction. Optical resonators for cancer biomarker detection use three configurations, as discussed earlier: microsphere, microring, and OFRR. There are both single resonator and array resonators. The system is usually a hybrid system involving microfluidics and LOCs. WGRs and ring resonators have better sensitivity and shorter response time than the other optical resonator configurations. Such advantages lead to the possibility of early detection for cancer diagnosis. Table 3 shows the relevant concentration of the example biomarkers which detected by optical resonator.

### 5.2. Optical Resonators for Nucleic Acid Testing (NAT)

Cancer can be detected by genetic biomarkers that can be produced from the development of the disease or the malfunctioning gene that causes the disease. The conventional methods of detecting nucleic acid-based molecules are heavily involved PCR to amplify the concentration of an analyte, resulting in better detection [28,129]. Such processes are time- and resource-consuming. Here, there is a trend for minimizing the PCR step or replacing it entirely [38,130]. The domain of interest is mainly the method for detecting the nucleic acid–based analyte in small volumes [17,20] extracted from both tumor cells and other body fluids. Researchers mainly compare analysis time with conventional PCR-based techniques as one of the dimensions of sensor quality. In fact, the goal of Nucleic Acid testing using optical resonator are to overcome the performance of PCR-based technology. Apart from seeking an advantage in sample preparation, there is also interest in developing less complex devices. The detected nucleic acid-based biomarkers are HER2 (human epidermal growth factor receptor [neu or ErbB2]) [138], HRAS (Harvey RAS) [131], FGFR3 [25], and gene abnormality, methylated genes [26,28], and mutated genes [129].

**Figure 9:**
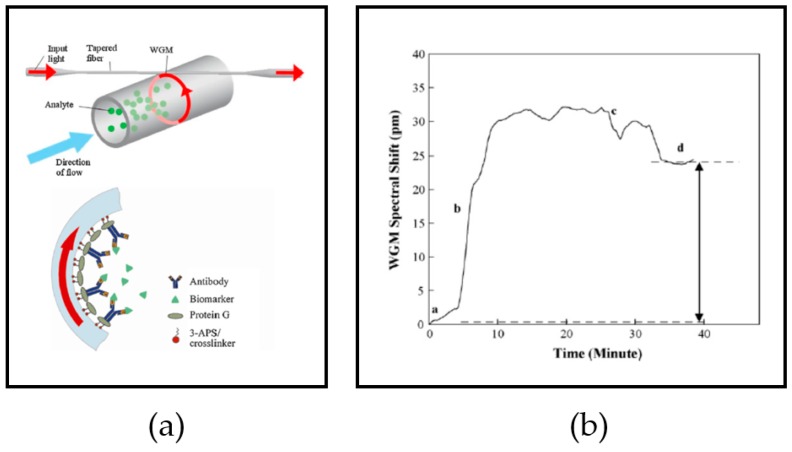
Analysis of HER2 biomarker. (**a**) OFRR schematic. The cross-section diagram visualizes the layer of the OFRR inner core surface; (**b**) spectral shift at (a) buffer flow, (b) sample flow, (c) sample binding and (d) buffer washes. Reprinted with permission from [138].

HER2 is overexpressed in the development of breast cancer. Presently, gene analysis involves a highly invasive method and labeled detection [11]. Therefore, less invasive and label-free analysis is gaining attention. HER2 is a transmembrane protein; its extracellular domain can also be detected in blood [15]. In 2010, an experiment was performed using an OFRR [138]. The resonator was a pulled SiO_2_ capillary with an outer diameter of 150 μm. The wall was chemically polished to reduce its thickness and to enhance sensitivity, and was reduced from 5 μm to 3 μm using hydrofluoric acid (HF). The resonator was coupled with a tapered SMF-28. The input laser had a wavelength of 1550 nm, with the photodetector on the other end of the tapered fiber. The sample was injected using a syringe pump at the rate of 1 μL/min. The inner core was functionalized first by a layer of aminosilane. Then, double mismatched primer (DMP) crosslinker bound protein G with the layer. HER2 antibody was introduced as the bioreceptor. The HER2 sample was diluted in phosphate-buffered saline (PBS) to 0.1 mg/mL. After the analysis, a low concentration of HF was applied to the inner surface to remove the activated surface component. The sensor was then ready to perform further diagnosis. Figure 9 shows the setting and the results.

Optical resonators were recently combined with the microfluidic technique to overcome the sample preparation process. Isothermal solid-phase amplification/detection (ISAD) was introduced. The concept usually involves a microring resonator combined with fluidic channel arrays. The technique is known for its high sensitivity, low LOD, and can be operated as a label-free sensor. The operation time is also reasonably short, and real**-**time analysis is possible. A 2013 study used ISAD on the *HRAS* and *FGR3* genes, which are bladder cancer biomarkers [131]. The performance of the ISAD device was compared with other multiplex analysis methods: isothermal recombinase polymerase amplification (RPA), conventional PCR, and real**-**time PCR (RT-PCR). Table 4 shows the results.

**Figure 10:**
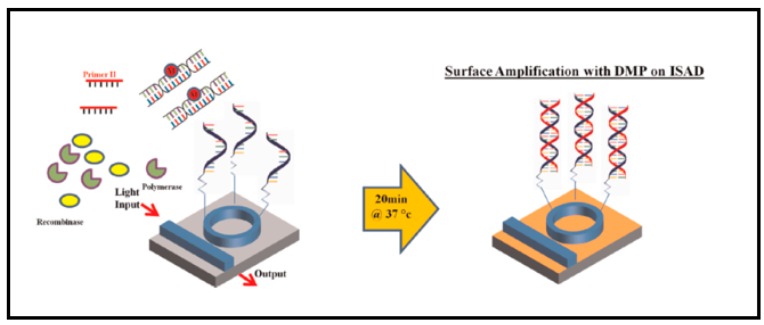
Diagram of *HRAS* and *FGR3* detection study. Surface functionalization on the microring resonator is visualized. Reprinted with permission from [131].

In 2014, Shin et al. [25] developed the ISAD system with DMP to improve specificity. Their aim was to detect mutated epidermal growth factor receptor (EGFR), the biomarker of non-small cell lung cancers (NSCLCs)**.** The sensor was fabricated similarly to a typical silicon microring resonator**.** Then, DMP primer was immobilized on the surface. The microring was incubated overnight in the amine-modified DMP primer in 1 PBS. A small acrylic chamber (1.5 cm × 0.7 cm × 2 cm) was used to confine the detection area (Figure 10).

### 5.3. Optical Resonators for Detecting Antigens

Antigen detection by optical resonators has focused on carcinoembryonic antigen (CEA) [24] and various carcinoma antigens (CAs). An antigen is the most detectable cancer biomarker (Table 2). In this field, microring resonators, OFRR, and microsphere resonators are utilized [27,137]. The existing methods for detecting antigen biomarkers are mainly based on commercialized ELISA platforms, which, as mentioned earlier, involve label-based detection. Antigens can be extracted from various biological sources: tumor cells, blood, and other fluids. Even though detecting an individual antigen yields insufficient information for diagnosing the type of cancer, it can benefit the prognosis for accurate treatment and early screening. For example, CEA is a biomarker of various cancers.

**Figure 11:**
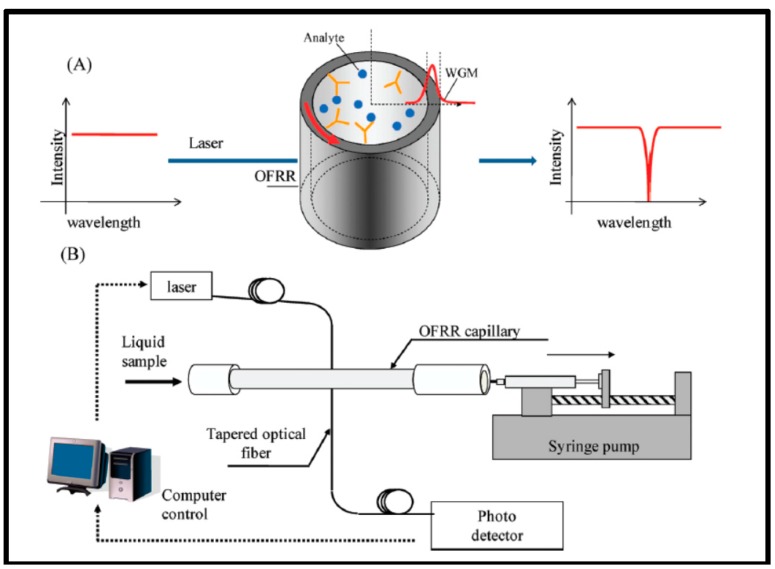
Schematic shows the surface functionalization of OFRR for detecting CA15-3; the system shows that the sample is drawn by the syringe pump while the OFRR performs the analysis. Reprinted with permission from [30].

In 2009, an OFRR was fabricated for detecting CA15-3 [30] (Figure 11), a breast cancer biomarker obtained from patient serum. The OFRR was pulled glass under high temperature from CO_2_ laser. A syringe pump and Tygon tubing were connected to the OFRR, which was then washed with HF solution to reduce the wall thickness. The tapered fiber was illuminated with a 980-nm laser. Then, anti-CA15-3 antibody was applied to the inner surface by amine coupling. First, the inner surface was treated with 50:50 hydrochloric acid (HCl)/methanol solution for 10 min and rinsed with DI water. The surface was aminated using 3-APS in ethanol. Next, the inner core was activated with 5% glutaraldehyde in PBS for 30 min Anti-CA15-3 (50 μg/mL) was introduced to the inner surface at a flow rate of 5 μL/min. To improve specificity, surface blocking of non-specific binding was crucial. In that regard, 1 mg/mL amine-PEG-amine in PBS was reacted with the surface for 30 min. The antibody-functionalized OFRR was then ready to detect the various concentrations of CA15-3 in PBS.

**Figure 12:**
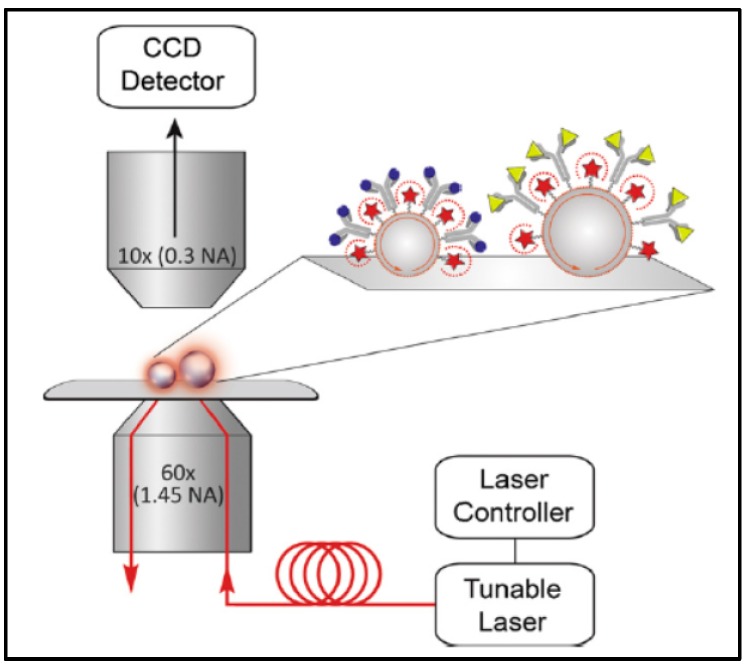
The multiple microsphere resonator system: the objective lens is utilized as the coupling waveguide, while two differently sized microspheres are paired with different receptors. CCD detects the fluorescence behavior of the system. Reprinted with permission from [136].

A microsphere configuration was used to detect CA125 and TNF-α [136]. The configuration was designed for detecting different analytes simultaneously. The experiment utilized the WGM Imaging (WGMI) technique. Sensitive fluorescent dye aided the imaging of the resonated microsphere, fluorescing only when the resonance condition was achieved. Instead of measuring the output light at the end of the coupling waveguide, the system detected the fluorescent ring on the microsphere surface as input tunable laser varying with wavelength. An image was obtained from a microscope above the microspheres. Figure 12 depicts the procedure.

**Figure 13:**
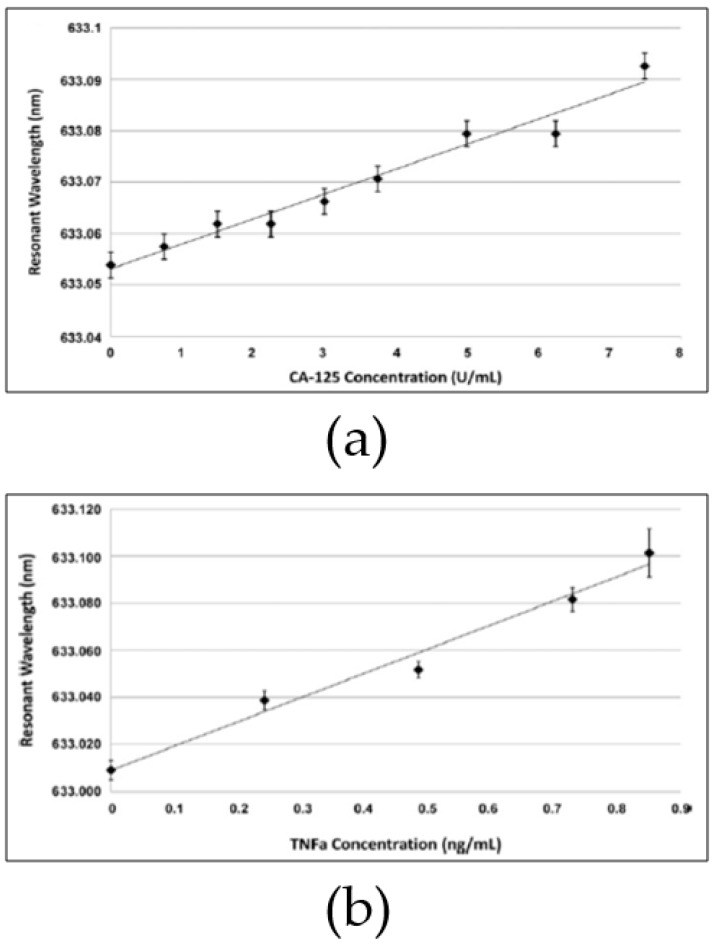
Calibration curves of microsphere performance. (**a**) CA-125 detection (38 μm) shows the linear relation between the applied concentration and the resonance wavelength; (**b**) TNF-α (53 μm) also shows the same relation. Reprinted with permission from [136].

Microspheres with two diameters were fabricated: 38 μm and 53 μm. The smaller sphere was designated as CA125 detector. Hence, it was incubated with 2 μg/mL anti-CA125. The bigger sphere was incubated with 2 μg/mL anti-TNF-α for detecting TNF-α. Both microspheres were fluoresced with commercial Alexa 633 dye. Then, surface blocking antigen was applied regularly. The experiment tested commercial CA-125 and TNF-α of known concentration. A high NA objective lens provided total internal reflection from the tunable laser source for evanescent coupling to occur and to enhance WGM. The system was then tested with known concentrations of samples; Figure 13 shows the results.

**Figure 14:**
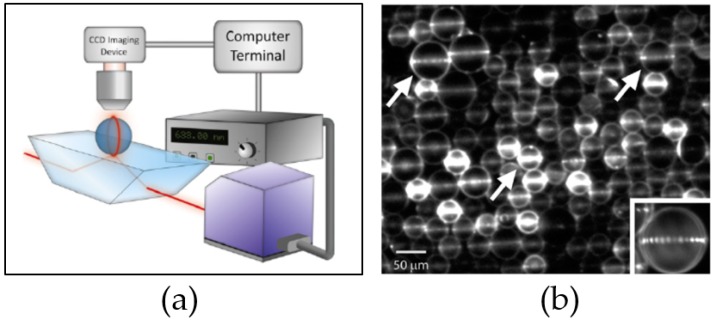
The configuration of ovarian cancer biomarker detection. (**a**) The system is based on a previous experiment but uses prism coupling for expanding the excitation area; (**b**) Fluorescence image revealing the illumination of some spheres specific for different markers. Reprinted with permission from [27].

Later on, the same research group examined the addition assay. Prism coupling was equipped as a WGM coupling waveguide for microspheres of three different diameters, which were designed as previously done for detecting different analytes (Figure 14) [27]. Three ovarian cancer biomarkers were investigated: osteopontin (38-μm microsphere), CA-125 (53-μm microsphere), and prolactin (63-μm microsphere). This allowed for 120 microspheres to be excited with WGM simultaneously, enabling the detection and real-time analysis of three components in the same assay.

### 5.4. Optical Resonators for Detecting Other Proteins

Apart from nucleic acid–based and antigen analytes, other biomarkers include proteins, enzymes, or other byproduct particles of cancer [31,139,140]. Optical resonators are also applied in many configurations to achieve the most suitable specification.

The enzyme telomerase is also a bladder cancer biomarker [135]. It is extracted from the cancer cell using heat shock. Urinary telomerase activity can lead to cancer detection. The conventional methodology is the telomerase repeat amplification protocol (TRAP), which is PCR**-**based and is time-consuming and costly.

**Figure 15:**
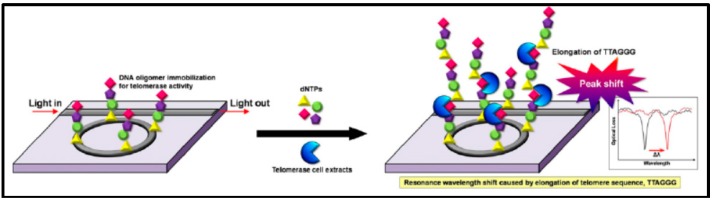
Diagram shows the functionalized surface of a microring resonator. DNA oligomer is immobilized on the surface. Telomerase extracted from a cancer cell is introduced into the system along with dNTPs. The result shows that the system can detect telomerase activity. Reprinted with permission from [38].

In 2013, an experiment analyzing telomerase activity with silicon microring resonators was performed [38]. A silicon microring of 4-μm diameter was fabricated with a 220 nm × 500 nm waveguide. The coupling length was 220 nm and the coupling waveguide–resonator gap was 220 nm (Figure 15).

**Figure 16:**
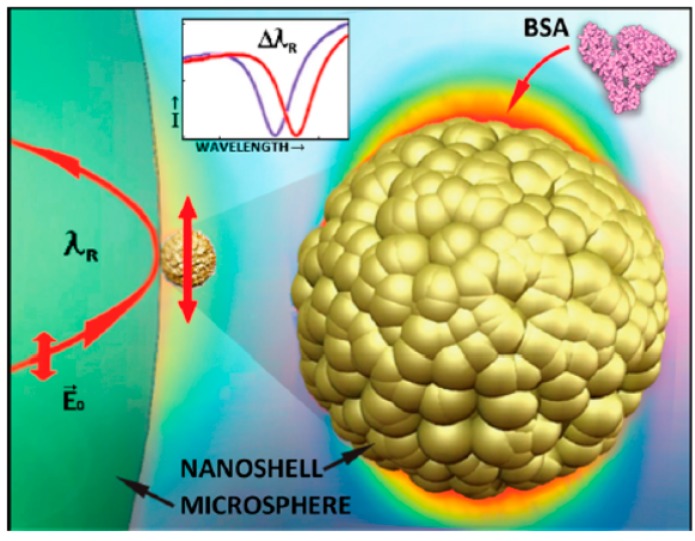
3D model shows the mechanism of BSA binding with gold nanoshell particle at the equator of a dielectric microsphere with WGM. The WGM-h enhancement and adsorption of the biomolecule caused the resonant wavelength shift. Reprinted with permission from [141].

The resonator was treated with oxygen plasma and soaked in 2% APTES (3-aminopropyltriethoxysilane) solution (in ethanol/DI water mixture) for 2 h in ambient conditions. The resonator was then heated and a mixture of GAD (glutamate decarboxylase) solution, borate buffer, and sodium cyanoborohydride was applied. The steps were repeated several times to prepare the surface. After surface activation, 50 μL telomerase oligomers solution was applied to the resonators and left overnight at 4 °C. Finally, the chip was placed in an acrylic chamber (6 × 2 × 1 mm^3^), and rinsed with 10 mM PBS to block the surface. Figure 16 shows the functionalized surface and binding mechanism. Detecting a single protein is also one of the challenges [29,111,112]. With nanotechnology, the detection of a single thyroid cancer biomarker has been reported for thyroglobulin protein (Tg) and bovine serum albumin (BSA). A dielectric microsphere was prepared with a single gold nanoshell bound at the equator (Figure 16). WGM inside the microsphere enhanced surface plasmon on the gold nanoshell, forming the hybrid system.

## 6. Conclusions

In this review, we discuss the principle of the optical resonator, provide a brief history thereof, and describe the application of WGM and ring resonators. Including the application of our interest, the biosensor, we also discuss the resonators and study in more detail the resonator types currently used in cancer detection. Lastly, we discuss cancer biomarkers, which mainly involve antigen or genetic components, and study and evaluate promising experiments and settings.

Optical resonators have the potential to be the future of cancer detection, as the technology is suitable for early-stage detection and effective prognosis. With early detection, a game-changer in healthcare industry, patients can be cured at the stage where cancer has not propagated to another site, which means more chances of successful treatment and less risk. With regard to effective prognosis, cancer prognostics at present are costly, time-consuming, and highly invasive. Optical resonators can detect analytes precisely in a shorter time, and as it involves biomarker testing, it requires less invasive procedures. These two benefits have resulted in the improvement of POC diagnosis, which will be the next generation of healthcare. In fact, microring resonators have been commercialized and their performance is impressive. As the manufacturer claims that the sensor can perform 128 tests simultaneously from a small sample, it involves the invasive extraction of blood from the patient.

In the present review, we divide the analysis into three categories based on biomarker group: Nucleic acid-based, antigen, and other protein components. Nucleic acid–based analytes are mainly genetic components or byproducts. The challenge is in improving throughput. Genetic samples are usually obtained in small amounts. PCR is mainly needed to generate sufficient quantities of analyte; however, it is time-consuming and expensive. Promising research has explored solutions for decreasing the reliance on PCR.

Antigens are the main cancer biomarkers; however, one antigen can be a biomarker of various cancers. In this application, specificity is the key factor, where one sample might contain many different antigen types. Nevertheless, the presence of a specific antigen can lead to a specific diagnosis. Hence, developing resonator quality for commercialization will be the new challenge. We also discuss protein and enzyme detection, with notable mention of an ultra-sensitive system capable of detecting individual proteins.

## Figures and Tables

**Table 1:** The relationship between the number system and TNM system of cancer for cervical cancer [7].

Number System	TNM System
Stage 0	Tis, N0, M0
Stage I	T1, N0, M0
Stage IA	T1a, N0, M0
Stage IB	T1b, N0, M0
Stage II	T2, N0, M0
Stage IIA	T2a, N0, M0
Stage III	T3, N0, M0
Stage IIIA	T3a, N0, M0
Stage IIIB	T3b, N0, M0 or T1–T3, N1, M0
Stage IV	-
Stage IVA	T4, N0, M0
Stage IVB	T1–T3, N0–N3, M1

**Table 2:** Known biomarkers according to type of tumor [17,24,135].

Tumor Type	Biomarker	Reference
Prostate	PSA, PAP	[17]
Testicular	α-Fetoprotein (AFP), β-human chorionic	[17]
Ovarian	CA125, AFP, hCG, p53, CEA	[17]
Breast	CA15-3, CA125, CA27.29, CEA BRCA1, CEA BRCA2, MUC-1, CEA, NY-BR-1, ING-1, HER2/NEU, ER/PR	[17]
Lung	AFP, CEA, EML4/ALK, EGFR, KRAS	[17,24]
Esophageal	SCC	[17,24]
Gastric	CA72-4, CEA, CA19-9	
Colon and pancreatic	CEA, CA19-9, CA24-2, p53, EGFR, KRAS, UGT1A1	[24]
Liver	AFP, CEA	[17]
Bladder	HRAS, FGFR3	[135]
Trophoblastic	SCC, hCG	[17]
Melanoma	Tyrosinase, NY-ESO-1, BRAF	[17]
Leukemia	Chromosomal abnormalities	[17]

**Table 3:** Known biomarker with clinical relevant concentration and reported detectable concentration with optical resonator.

Biomarker	Clinical Relevant Concentration	Type of Optical Resonator	Detection Limit	Time (min)	Ref.
CA-125	35 U/mL	Microsphere resonator	∼1.5 U/mL	-	[136]
CA-15-3	30 U/mL	OFRR	1 U/mL	20	[30]
EGFR Mutation	25% of mutant allele	Microring resonator	1% of mutant allele	20	[25]
TNF-α	203 pg/mL	Microsphere resonator	∼240 pg/mL	-	[136]
CEA	3–5 ng/mL	Microring resonator	2 ng/mL	30	[137]
HER2	15 ng/mL	OFRR	1 ng/mL	15–30	[138]

**Table 4:** Comparison of LOD, analysis time, and label of various methods [131].

Parameter	ISAD	RPA	PCR	RT-PCR
LOD	500 fg/μL	50 pg/μL	50 pg/μL	5 pg/μL
Analysis time (min)	20–30	40–50	120–180	60–120
Label	Label-free	EtBr	EtBr	Fluorescence

## Abbreviations

POC	Point-of-care
WGM	Whispering-gallery mode
Q-factor	Quality factor
WGR	WGM resonator
CW-WGM	Continuous-wave WGM
RIU	Refractive index unit
LOD	Limit of detection
UV	Ultraviolet
CMOS	Complementary metal-oxide semiconductor
DUV	Deep UV lithography
NIL	Nanoimprinting lithography
3D	Three-dimensional
OFRR	Optofluidic ring resonator
μTAS	Micro total analysis system
LOC	Lab-on-a-chip
PCR	Polymerase chain reaction
FDA	Food and Drug Administration
ELISA	Enzyme-linked immunosorbent assay
EtBr	Ethidium bromide
RIA	Radioimmunoassay
NAT	Nucleic acid testing
ISAD	Isothermal solid-phase amplification/detection
RPA	Recombinase polymerase amplification
WGMI	WGM imaging
TRAP	Telomerase repeat amplification protocol
APTES	3-Aminopropyltriethoxysilane
PBS	Phosphate-buffered saline
GAD	Glutamate decarboxylase
BSA	Bovine serum albumin
PSA	Prostate-specific antigen
PAP	Papanicolaou
AFP	Alpha-fetoprotein
CA125	Cancer antigen 125
hCG	Human chorionic gonadotropin
CEA	Carcinoembryonic antigen
CA15-3	Cancer antigen 15-3
CA27.29	Cancer antigen 27-29
MUC-1	Mucin 1
ING-1	Inhibitor of growth protein 1
HER2/neu	Human epidermal growth factor receptor 2
ER	Estrogen receptors
